# A Characterization of Aerosolized Sudan Virus Infection in African Green Monkeys, Cynomolgus Macaques, and Rhesus Macaques

**DOI:** 10.3390/v4102115

**Published:** 2012-10-15

**Authors:** Elizabeth E. Zumbrun, Holly A. Bloomfield, John M. Dye, Ty C. Hunter, Paul A. Dabisch, Nicole L. Garza, Nicholas R. Bramel, Reese J. Baker, Roger D. Williams, Donald K. Nichols, Aysegul Nalca

**Affiliations:** 1 Center for Aerobiological Sciences, U.S. Army Medical Research Institute of Infectious Diseases (USAMRIID), 1425 Porter Street, Fort Detrick, Maryland 21702, USA; 2 Virology Division, USAMRIID, 1425 Porter Street, Fort Detrick, Maryland 21702, USA; 3 Toxicology Division, USAMRIID, 1425 Porter Street, Fort Detrick, Maryland 21702, USA; 4 Pathology Division, USAMRIID, 1425 Porter Street, Fort Detrick, Maryland 21702, USA

**Keywords:** filovirus, ebolavirus, aerosol, primate, animal model

## Abstract

Filoviruses are members of the genera *Ebolavirus*, *Marburgvirus*, and “Cuevavirus”*. *Because they cause human disease with high lethality and could potentially be used as a bioweapon, these viruses are classified as CDC Category A Bioterrorism Agents. Filoviruses are relatively stable in aerosols, retain virulence after lyophilization, and can be present on contaminated surfaces for extended periods of time. This study explores the characteristics of aerosolized Sudan virus (SUDV) Boniface in non-human primates (NHP) belonging to three different species. Groups of cynomolgus macaques (cyno), rhesus macaques (rhesus), and African green monkeys (AGM) were challenged with target doses of 50 or 500 plaque-forming units (pfu) of aerosolized SUDV. Exposure to either viral dose resulted in increased body temperatures in all three NHP species beginning on days 4–5 post-exposure. Other clinical findings for all three NHP species included leukocytosis, thrombocytopenia, anorexia, dehydration, and lymphadenopathy. Disease in all of the NHPs was severe beginning on day 6 post-exposure, and all animals except one surviving rhesus macaque were euthanized by day 14. Serum alanine transaminase (ALT) and aspartate transaminase (AST) concentrations were elevated during the course of disease in all three species; however, AGMs had significantly higher ALT and AST concentrations than cynos and rhesus. While all three species had detectable viral load by days 3-4 post exposure, Rhesus had lower average peak viral load than cynos or AGMs. Overall, the results indicate that the disease course after exposure to aerosolized SUDV is similar for all three species of NHP.

## 1. Introduction

The members of the family *Filoviridae*, first identified in 1967, are filamentous, enveloped, non-segmented, negative-sense RNA viruses with 19 kb genomes encoding seven structural gene products [1]. These include marburgviruses and ebolavirus. The natural reservoirs of these negative-strand RNA viruses are thought to be various African fruit bats [2]. Two marburgviruses, Marburg virus (MARV) and Ravn virus (RAVV) and four ebolaviruses, Bundibugyo virus (BDBV), Ebola virus (EBOV), Sudan virus (SUDV), and Taï Forest virus (TAFV) cause human disease [3,4,5]. Filoviruses cause severe hemorrhagic fever in humans and non-human primates (NHP), causing epidemics at times involving hundreds of people. Filoviruses, which are classified as Category A Bioterrorism Agents by the Centers for Disease Control and Prevention (Atlanta, GA), have stability in aerosol form comparable to other lipid containing viruses such as influenza A virus, a low infectious dose (<10 pfu) by the aerosol route in NHPs, and case fatality rates as high as ~90% ([6,7,8]). There are allegations that the former Soviet Union weaponized filoviruses [9]. Filoviruses in aerosol form are therefore considered a possible serious threat to the health and safety of the public. A number of promising vaccines and post-exposure treatments have been developed but are not yet available [10]. However, the efficacy of most of these vaccines and therapies has not been determined for an aerosol route of viral challenge (exceptions include [11,12]). 

The mode of acquisition of viral infection in index cases is usually unknown. Secondary transmission of filovirus infection is typically thought to occur by direct contact with infected persons or infected blood or tissues. There is no strong evidence of secondary transmission by the aerosol route in African filovirus outbreaks. However, aerosol transmission is thought to be possible and may occur in conditions of lower temperature and humidity which may not have been factors in outbreaks in warmer climates [13]. At the very least, the potential exists for aerosol transmission, given that virus is detected in bodily secretions, the pulmonary alveolar interstitial cells, and within lung spaces [14]. 

Filovirus infection models have been developed in mice, hamsters, guinea pigs, pigs, and NHPs [15,16,17]. Of these models however, only pigs and NHPs can be infected without virus adaptation. NHPs have demonstrated, sudden onset of fever, clinical signs involving multiple organ systems (gastrointestinal, respiratory, neurological, and vascular), liver dysfunction, coagulopathy resulting in hypovolemic shock, multi-organ system failure, and death. The typical route of inoculation for the animal models has been intramuscular (i.m.) or intraperitoneal (i.p.), despite a perceived threat by the aerosol route. Thus, well-characterized NHP models of aerosolized filovirus infections are needed for testing vaccines and therapeutics to satisfy the fulfillment of the Food and Drug Administration’s “Animal Rule” [18].

Intentional direct aerosol exposure resulting in productive infection of NHPs with filoviruses has been demonstrated in a number of studies. These studies include aerosol infection of rhesus macaques (rhesus), African green monkeys (AGMs), and cynomolgus macaques (cynos) with MARV and EBOV; and vaccine studies testing vesicular stomatitis virus (VSV) or adenovirus-based vaccines against filovirus aerosol challenge of rhesus and cynos [8,11,12,13,19,20,21,22]. In one study, rhesus infected by the aerosol route with EBOV had virus detected first in the lungs and bronchial washings (day 2), followed by the blood and liver (day 3) [20]. Notably, infection of rhesus with EBOV by the aerosol route resulted in lesions consistent with those seen in i.p. infections. However, the animals that were infected with aerosolized virus also had lung lesions with a bronchocentric pattern and the heaviest antigen concentration in lung tissue adjacent to the bronchioles [13]. 

The only published study utilizing aerosolized SUDV in NHPs demonstrated protection of cynos against aerosolized SUDV using a complex adenovirus based vector (CAdVax) vaccine system [11]. An important finding of this study is that aerosolized SUDV is more likely than aerosolized EBOV to result in hemorrhagic pneumonia. Despite this, characterization of a NHP model of infection with aerosolized SUDV has not been reported. To this end, we exposed rhesus, cynos, and AGMs, to SUDV by the aerosol route, and monitored the clinical course of the disease. Three NHP species were chosen for this study for comparison, since each was previously used to characterize the disease course and vaccine and therapeutic efficacy tests by other routes of filovirus exposure. A primary goal of this study was to determine what differences, if any, exist in the clinical disease progression following airborne SUDV infection of these three NHP species. Clinical disease, temperature, heart rate, pressure, complete blood counts (CBC), blood chemistry, and viral load were monitored daily and the results of these observations are reported here. This report represents the first detailed characterization of the clinical disease course caused by aerosolized SUDV in rhesus, cynos, and AGMs.

## 2. Results and Discussion

### 2.1. Aerosol Exposure

Rhesus, cynos, and AGMs were exposed to either low (50 pfu) or high (500 pfu) target doses of aerosolized SUDV Boniface. This study was divided into six tiers, performed sequentially, according to dose and primate species. The actual doses delivered were determined based on plaque assay of the contents of the AGI aerosol sampling device and are detailed in Table 1. For each NHP species, the actual viral doses that the animals in the 50 pfu target dose group received were within 25% of the target dose. The average actual dose for the 500 pfu groups was close to the target dose for AGMs and rhesus but for cynos was less than one-third of the intended dose. This lower-than-expected “high” target dose may have minimized the dose-dependent differences in disease parameters, particularly survival times, between the high and low dose groups of cynos compared to those of AGMs and rhesus. 

**Table 1:** Summary of outcome of aerosolized Sudan virus (SUDV) exposure in non-human primates (NHPs).

NHP	Target Dose (pfu)	Actual Dose (pfu +/- SEM)	# Survived/ # Exposed	Survival (days +/- SEM)	# with Rash/# Exposed	Rash onset (Ave. days +/- SEM)	# with Dyspnea/# Exposed	Dyspnea (Ave. days +/- SEM)
**AGM**	50	66.3 ± 15.5	0/6	8.8 ± 0.4	3/6	8.0 ± 2.0	6/6	7.2 ± 0.8
**AGM**	500	363.9 ± 64.5	0/6	8.5 ± 0.4	3/6	8.6 ± 0.3	6/6	5.0 ± 0.4
**Cyno**	50	44.6 ± 10.4	0/6	7.8 ± 0.5	6/6	6.3 ± 0.3	5/6	6.0 ± 0.8
**Cyno**	500	131.5 ± 28.5	0/6	7.5 ± 0.2	6/6	6.0 ± 0.5	6/6	7.2 ± 0.9
**Rhesus**	50	50.0 ± 7.4	1/6	10.0 ± 0.3	4/6	8.5 ± 0.6	6/6	6.0 ± 0.8
**Rhesus**	500	345.2 ± 18.1	0/6	8.5 ± 0.6	2/6	6.5 ± 0.5	6/6	6.2 ± 0.3

### 2.2. Survival

**Figure 1:**
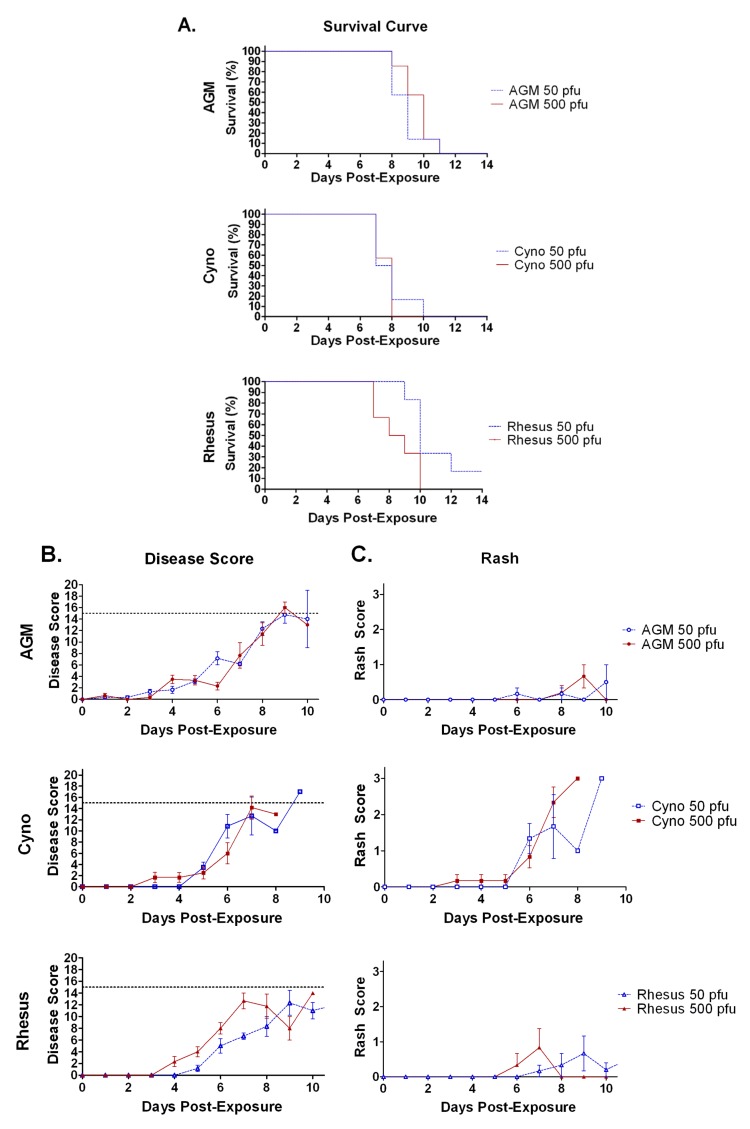
Survival, clinical disease scores, and rash in NHPs exposed to aerosolized SUDV. **A**) Percent survival of NHP exposed via two different doses of aerosolized SUDV. **B**) Average clinical disease scores and **C**) average rash score over time after exposure of three different species of NHP to aerosolized SUDV (Figures B and C share a legend). There were six animals per group. Error bars represent the standard error of the mean. The dashed line on figure B represents the cutoff score, above which, animals were humanely euthanized.

Survival curves are shown in Figure 1A. All animals from all groups met the criteria for euthanasia between days 6 to 12 post-exposure to aerosolized SUDV, except for one of six rhesus exposed to 50 pfu. The lone rhesus survivor became ill, but recovered (Table 1). The shortest mean survival occurred in the cynos, with average survival times of 7.8 and 7.5 days for 50 and 500 pfu groups, respectively. Overall, the survival time of cynos was significantly shorter than either AGMs (*p *= 0.002) or rhesus (*p = *0.0003). The AGMs had an intermediate survival time post-exposure, with 50 and 500 pfu groups surviving for an average of 8.8 and 8.5 days. Rhesus had the longest survival with 10.0 and 8.5 day average survival times post-exposure to 50 or 500 pfu. Data from only the five rhesus that died in the low-dose group were used in calculating survival times.

### 2.3. Clinical Signs

Signs of disease in all groups included fever, reduced responsiveness/depression, petechial rash, reduced urine and fecal output, reduced appetite, labored breathing, and dehydration. Overall, the severity of the clinical disease increased with time and all NHP groups had significantly increased clinical disease scores late in infection by student’s t-tests (Figure 1B). The criteria for evaluation of clinical disease are described in Section 3.6.

The onset of clinical signs was earliest in the AGMs, with some animals (four of six in each group) appearing dehydrated from days 1 to 3 post-exposure and some animals (one of six for the 50 pfu group and three of six for the 500 pfu group) with dyspnea on day 4 post-exposure. Cynos in the 500 pfu target dose group began showing clinical signs of disease on day 3 with signs such as fever (one of six), dyspnea (one of six), and reduced appetite (one of six). In contrast, cynos in the 50 pfu exposure group did not begin to show clinical signs until day 4 with fever in two of six animals. Rhesus exposed to a 50 pfu target dose did not develop clinical signs until day 5 with an onset of fever in three of six animals. Rhesus in the 500 pfu group developed clinical signs on day 4, with onset of dyspnea (three of six), reduced stool (two of six), dehydration and fever (one of six). 

Weight loss of more than 10% occurred in only one animal, an AGM in the 50 pfu exposure group (data not shown). Bleeding from the nose, rectum, or venipuncture site was observed in two animals from the 50 pfu rhesus group, three animals in the 500 pfu cyno group and one animal in the 50 pfu cyno group. In contrast, dyspnea was observed in all but one of the animals during the disease course, with an average onset between 5.0–7.2 days post-exposure to aerosolized SUDV (Table 1). 

Cutaneous petechiae (also known as a “petechial rash”) were observed earliest and most frequently in cynos, with an onset as early as three days post-exposure and all animals from both exposure groups eventually developing the rash (Figure 1C and Table 1). Rash was not a uniform occurrence in exposed AGMs and rhesus, with a frequency of 33–67%. AGMs with rash, had only minor rash scoring no greater than 1 (barely visible), typically when the animals were moribund. Rashes in AGMs, not a usual occurrence according to published filovirus studies, were observed in armpits, inner arm, or on the side of chest. 

### 2.4. Fever

**Figure 2:**
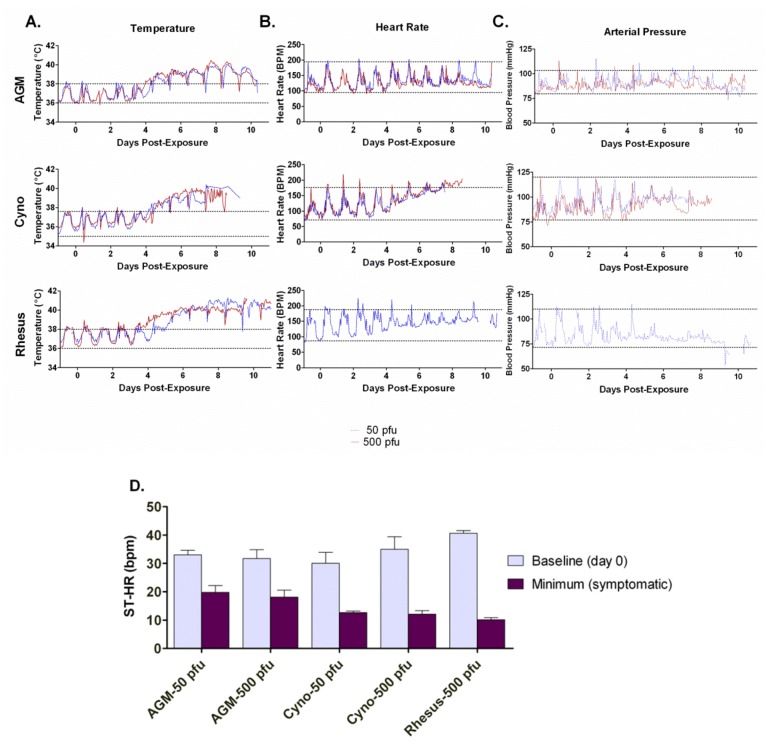
Telemetry: Temperature, Heart Rate, Arterial Pressure. **A**) Temperature, **B**) heart rate and **C**) pressure were measured by internally implanted telemetry devices once every hour throughout the study in NHPs exposed to two different doses of aerosolized SUDV. There were six animals per group. The values shown represent the average for each group. The straight dashed lines indicate the baseline maximum and minimum average diurnal values before exposure. **D**) The standard deviation of the heart rate (SD-HR) was calculated, expressed as beats per minute (bpm). For all species and doses examined, the SD-HR decreased significantly post-exposure, suggesting a loss of the diurnal rhythm (*p *<0.01). Telemetry was not calculated for the rhesus 50 pfu group due to equipment failure.

Fever was defined as the time at which temperature increased greater than 1.5 °C above the peak diurnal baseline established before exposure (Figure 2A). The average onset of fever in AGMs was 4.0 days for the 50 pfu exposure group and 3.3 days for the 500 pfu exposure group (Table 2). Cynos had an average onset of fever at 4.2 days for the 50 pfu group and 4.7 days for the 500 pfu group. Rhesus in the 50 and 500 pfu groups had an onset of 4.4 and 3.3 days, respectively. There were no significant differences among groups in fever onset, maximum temperature, fever duration, or fever hours.

**Table 2:** Summary of fever data.

NHP	Target Dose (pfu)	Fever Onset^a^	ΔT_max_, °C^b^	Avg Elevation, °C	Fever Hours^c^
**AGM**	50	4.0	4.9	3.8	224.0
**AGM**	500	3.3	4.0	3.0	184.3
**Cyno**	50	4.2	6.3	4.8	221.8
**Cyno**	500	4.7	5.1	3.9	191.5
**Rhesus**	50	4.4	4.6	3.0	182.1
**Rhesus**	500	3.3	4.4	3.1	254.3

^a^: Defined as the first day after viral challenge with 8 or more hours of significant temperature elevation (as determined by ARIMA modeling). **^b^**:ΔT_max_ is the maximum change in temperature. ^c^: Calculated as the sum of the significant temperature elevations. ^d^: *p *>0.05 between all groups for fever onset, ΔT_max_, fever duration, and fever hours.

### 2.5. Heart Rate and Blood Pressure

At approximately the same time as the onset of fever, the normal diurnal changes in heart rate were disrupted and heart rate increased overall (Figure 2B). This phenomenon was particularly pronounced in cynos but not as evident in the AGMs. The SD-HR was similar across all three species during the baseline pre-exposure recording (Figure 2D). After exposure, the SD-HR significantly decreased in all species and at all doses, reaching a minimum between approximately 5 and 8 days post-exposure (AGM 50 pfu, *p = *0.0012; AGM 500 pfu, *p = *0.0012; cyno 50 pfu, *p = *0.0072, cyno 500 pfu, *p = *0.0018; rhesus 500 pfu, *p *<0.0001). SD-HR was not calculated for the rhesus 50 pfu group, since heart rate and pressure data was not collected for this group due to equipment failure. The number of days to reach the minimum SD-HR were significantly less than the number of days to meeting criteria for euthanasia for all groups (*p *<0.05). A disruption of the diurnal variation in blood pressure was also seen in all three primate species upon exposure to aerosolized SUDV (Figure 2C). Similar to the alteration in heart rate, these changes coincided with the onset of fever. The most pronounced changes were in cynos and rhesus with a trend in all three groups for reduced pressure readings as the infection progressed. 

### 2.6. Cell Counts

**Figure 3:**
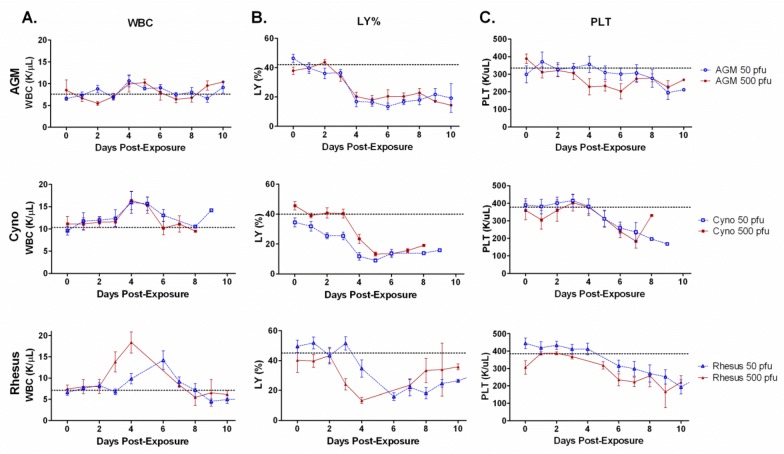
**Average white blood cell count (WBC), percentage of lymphocytes and platelet number.** Graphs are shown for the group averages of **A**) WBC, **B**) lymphocytes, **C**) platelets in three NHP species after exposure to two different doses of aerosolized SUDV. Average measurements on day 0 were taken before exposure serve as a baseline. There were six animals per group. Error bars represent the standard error of the mean. The horizontal dashed line indicates the average baseline (day 0) for the 12 animals in both dosage groups. WBC increased significantly above baseline for cynos and rhesus (*p*<0.05). All groups had significant decrease in lymphocytes late in infection (*p*<0.01) except the rhesus 500 pfu group (*p *>0.05). Platelets decreases were significant for cyno, AGM 500 pfu and rhesus 50 pfu groups (*p *<0.01).

White blood cell (WBC) concentrations increased in all animals, peaking on day 4 post-exposure in each group except for the 50 pfu rhesus group, which had a peak on day 6 (Figure 3A). The increase at the peak concentration was significant by a two-tailed paired t-test for the 50 and 500 pfu cynos groups (*p =* 0.02 and *p = *0.01) and rhesus 50 and 500 pfu groups (*p = *0.03 and *p = *0.006), but not for AGMs in 50 or 500 pfu exposure groups (*p = *0.1 and *p = *0.3). After this peak, average WBC concentrations generally decreased until the animals reached the criteria for euthanasia. 

In this study, the average percentage of lymphocytes decreased in each of the groups after exposure to aerosolized SUDV (Figure 3B). These decreases were caused by reductions in the absolute number of lymphocytes combined with increases in other populations of white blood cells (data not shown). The decreases in lymphocyte percentage were significant in all groups late in the disease course, except for the rhesus group exposed to 500 pfu (AGM 50 pfu, *p = *0.01; AGM 500 pfu, *p = *0.02; cyno 50 pfu, *p = *0.006; cyno 500 pfu, *p = *0.0004; rhesus 50 pfu, *p = *0.0004; rhesus 500 pfu, *p = *0.14). These concentrations stayed depressed throughout the remainder of the disease course for AGMs and cynos. In contrast, lymphocyte percentage concentrations in both rhesus groups rebounded partially before animals met criteria for humane euthanasia, and the lone surviving animal in the 50 pfu exposure group had lymphocyte percentage values that returned to baseline concentrations. 

Likewise, in this study, exposure to aerosolized SUDV resulted in a steady decrease in platelets in all groups throughout the course of the disease (Figure 3C). By the latter part of the disease, these decreases were significant in AGMs exposed to 500 pfu (*p = *0.002), cynos exposed to 50 pfu or 500 pfu ( *p =* 0.004 and *p = *0.006), and rhesus exposed to 50 pfu (*p = *0.006), but not in AGMs exposed to 50 pfu or rhesus exposed to 500 pfu (*p *>0.05).

### 2.7. Chemistries

**Figure 4:**
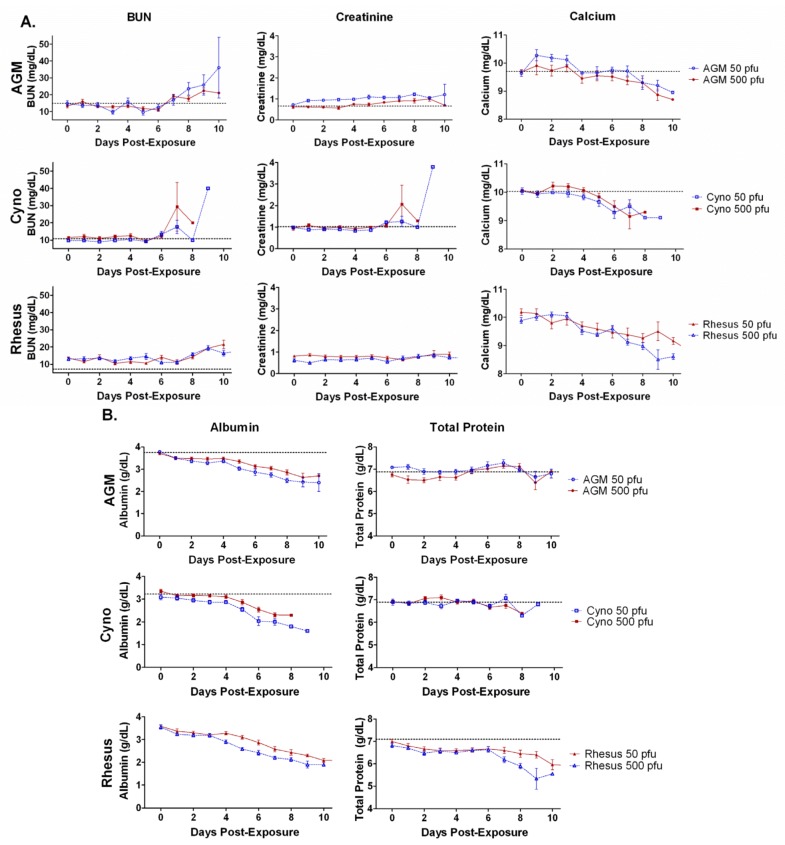
Blood urea nitrogen (BUN), creatinine, total calcium, albumin, and total protein averages. Graphs are shown for the group averages of **A**) BUN, creatinine, and total calcium, and **B**) albumin and total protein in three NHP species after exposure to two different doses of aerosolized SUDV. Average measurements on day 0 were taken before exposure serve as a baseline. There were six animals per group. Error bars represent the standard error of the mean. The horizontal dashed line indicates the average baseline (day 0) for the 12 animals in both dosage groups. BUN increases were significant for African green monkeys (AGM), rhesus 50 pfu, and cyno 50 pfu groups (*p *<0.05). Creatine increases were significant for AGM groups and rhesus 50 pfu groups (*p *<0.001). Calcium decreases were significant for all groups (*p *<0.01) except the AGM 50 pfu group. Albumin decreases were significant for all groups (*p *<0.01). Decreases in protein were significant for all groups (*p *≤0.01) except for AGM 500 pfu and cyno 50 pfu groups.

Creatinine and blood urea nitrogen (BUN) were measured to assess renal function (Figure 4A). BUN concentrations increased late in infection in all groups. Average BUN concentrations had increased significantly late in infection in AGMs exposed to 50 or 500 pfu (*p =* 0.02 and *p = *0.0007) and rhesus and cynos exposed to 50 pfu (*p = *0.02 and *p = *0.03), but were not significant in rhesus or cynos exposed to 500 pfu (*p *>0.05). Creatinine concentrations increased slightly, and significantly in AGMs exposed to 50 or 500 pfu (*p = *0.0002 and *p = *0.0007) and rhesus exposed to 50 pfu (*p = *0.0009), but the increase was not statistically significant in the other three groups (*p *>0.05). 

Average total calcium concentrations decreased steadily throughout the disease course for each group, with significant decreases in all groups late in infection, except for AGMs exposed to 50 pfu (AGM 50 pfu, *p* >0.05; AGM 500 pfu, *p = *0.005; cynos 50 pfu, *p = *0.007; cynos 500 pfu, *p = *0.02; rhesus 50 pfu, *p = *0.006; rhesus 500 pfu, *p = *0.007). 

Hypoalbuminemia gradually developed in each of the three species and for each exposure dose (Figure 4B). The decrease was significant for all groups late in infection (AGM 50 pfu, *p*<0.0001; AGM 500 pfu, *p = *0.004; cyno 50 pfu, *p = *0.007; cyno 500 pfu, *p*<0.0001; rhesus 50 pfu, *p*<0.0001; rhesus 500 pfu, *p*<0.0001). While changes in total protein concentrations were less pronounced (Figure 4B), rhesus in particular showed a trend towards decreasing total protein concentrations, with greater decreases than those of cynos or AGMs throughout the disease course (p<0.05). Decreases in total protein were significant in AGMs exposed to 50 pfu (*p = *0.0007), cynos exposed to 500 pfu (*p = *0.003), and rhesus exposed to 50 or 500 pfu (*p = *0.01 and *p = *0.007). AGMs exposed to 500 pfu and cynos exposed to 50 pfu did not have significant decreases (*p *>0.05), keeping more consistent concentrations of total protein throughout the study. 

**Figure 5:**
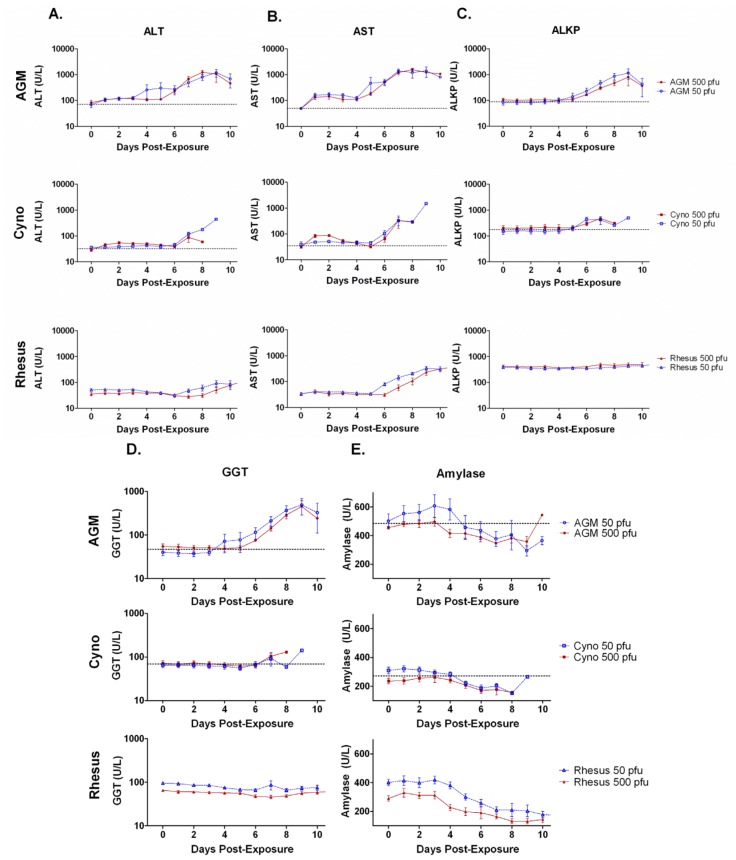
Average alanine transaminase (ALT), aspartate transaminase (AST), alkaline phosphatase (ALKP), gamma-glutamyltransferase (GGT), and amylase concentrations in NHPs exposed to aerosolized SUDV. Graphs are shown for the group averages of **A**) ALT, **B**) AST, **C**) ALKP, **D**) GGT, and **E**) amylase in three NHP species after exposure to two different doses of aerosolized SUDV. Average measurements on day 0 were taken before exposure serve as a baseline. There were six animals per group. Error bars represent the standard error of the mean. The horizontal dashed line indicates the average baseline (day 0) for the 12 animals in both dosage groups. AGM groups had significant changes in all parameters (*p *≤0.01) except the AGM 50 pfu group for amylase. Cyno groups had significant increases primarily in ALKP (*p *≤0.01) and the 50 pfu group for amylase (*p *<0.01). Rhesus groups had significant changes in AST and amylase (*p *≤0.01) but not for other parameters.

Alanine transaminase (ALT), aspartate transaminase (AST), alkaline phosphatase (ALKP), and gamma-glutamyltransferase (GGT) were evaluated throughout the disease course to assess the degree of liver damage (Figure 5 A-D). AGMs exposed to 50 or 500 pfu had pronounced increases in ALT (*p =* 0.01 and *p = *0.01), AST (*p= *0.0003 and *p = *0.009), ALKP (*p = *0.02 and *p = *0.003), and GGT (*p = *0.02 and *p = *0.006). Cynos exposed to 50 or 500 pfu had several significant increases during the later part of the disease, with AST (*p = *0.04 and *p* >0.05), ALKP (*p = *0.002 and *p = *0.01), and GGT (*p = *0.046 and *p = *0.3) and no significant increase in ALT for either dosage group. In contrast, rhesus from 50 and 500 pfu exposure groups did not have significant increases in ALT, ALKP, or GGT, but did have significant increases in AST late in the disease course (*p = *0.01 and *p = *0.01). 

Amylase was also tested (Figure 5E). Average amylase concentrations decreased for each group, with significant decreases for AGMs exposed to 500 pfu (*p = *0.005), cynos exposed to 50 pfu (*p = *0.006) and rhesus from both exposure groups (*p =* 0.003 and *p = *0.002). Decreases in amylase in AGMs or cynos exposed to 50 or 500 pfu, were not significant (*p* >0.05). Total bilirubin increased only slightly and these increases were only significant in cyno 500 pfu and AGM 50 pfu groups (data not shown). 

### 2.8. Viral Load

**Figure 6:**
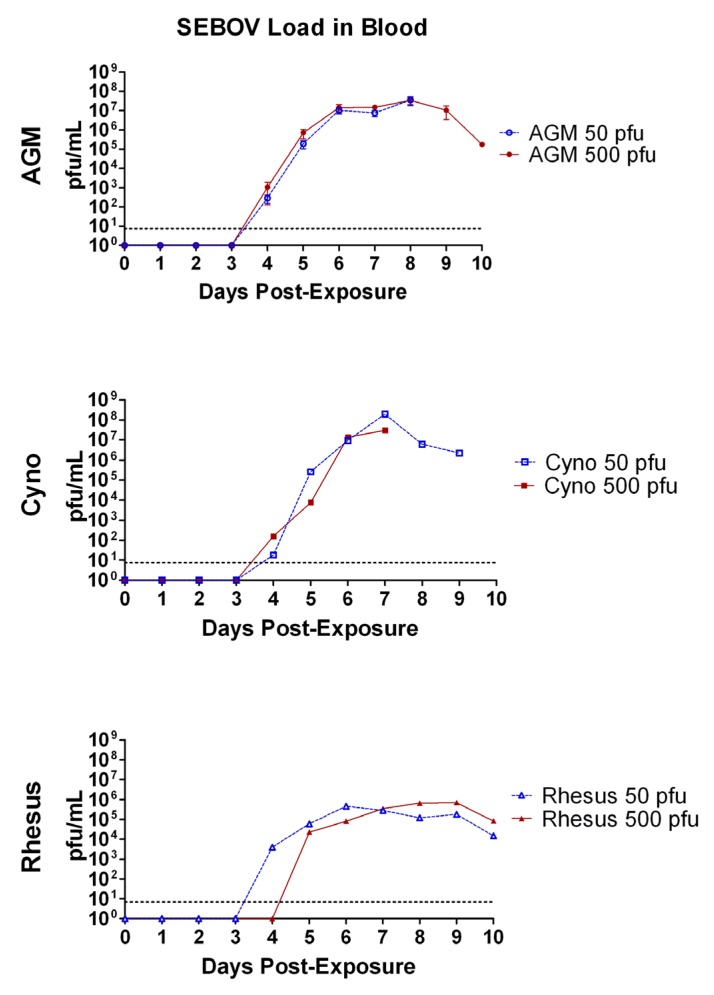
Average SUDV viral load in blood samples drawn daily from NHPs exposed to aerosolized SUDV. SUDV genomes were quantified by real-time RT-PCR and a standard curve was used to report the results in terms of pfu/mL. Graphs are shown for group averages of SUDV pfu/mL of blood drawn each day on the day of (day 0) and following exposure to aerosolized SUDV. There were six animals per group. Error bars represent the standard error of the mean. Rhesus peak viral load was significantly less than AGMs (*p* <0.01) but not cynos.

Real-time RT-PCR to detect and quantify SUDV was performed on daily blood samples from each animal in the study (Figure 6). The assay was previously shown to detect as little as 0.1 pfu per reaction [23]. SUDV was detected in the blood on day 4 for all groups except for the rhesus 50 pfu group, in which virus was first detected on day 5. The average peak concentration of viral load was at least 10^7.5^ pfu/mL for AGMs and cynos of both exposure groups. Interestingly, for both groups of rhesus, the average peak concentration was approximately 5x10^5^ pfu/mL, which was significantly less than AGMs (*p = *0.003) and cynos (*p = *0.007). The day of the average peak also varied. The earliest peak occurred in the rhesus 50 pfu exposure group (day 6), followed by both cyno groups (day 7), both AGM groups (day 8), and the rhesus 500 pfu group. In animals surviving beyond the peak day of viral load, concentrations detected in the blood dropped slightly. The blood viral load in the single rhesus that survived the infection peaked at day 7 post exposure with a concentration of 10^5 ^pfu/mL. 

## 3. Experimental Section

### 3.1. Animals

Healthy adult African green monkeys (*Chlorocebus aethiops*), cynomolgus macaques (*Macaca fascicularis*), and rhesus macaques (*Macaca mulatta*) of both sexes were obtained from the NHP colony at the United States Army Medical Research Institute of Infectious Diseases (USAMRIID). Baseline weights were between 3.5-5.2 kg for AGMs, 5.8–8.2 kg for cynos and 3.6–5 kg for rhesus. All NHPs were surgically implanted with subcutaneous PhysioTel D-70-PCT telemetry devices (Model # TL11M2-D70-PCT), Data Sciences International, St. Paul, MN) and allowed to recover for at least 45 days before being transferred to biosafety level 4 (BSL-4) containment facilities. Animals were acclimated to the BSL-4 containment facility for at least 1 week before virus exposure and baseline telemetry data were collected during this acclimation period. 

### 3.2. Ethics and Animal Welfare

Research was conducted in compliance with the Animal Welfare Act and other federal statutes and regulations relating to animals and experiments involving animals, and adhered to the principles stated in the Guide for the Care and Use of Laboratory Animals (National Research Council, 2011). The facility where this research was conducted (USAMRIID) is fully accredited by the Association for the Assessment and Accreditation of Laboratory Animal Care International (Frederick, MD). Research was conducted under a protocol approved by the Institutional Animal Care and Use Committee (IACUC) at USAMRIID. Animals were provided fresh fruits, vegetables, toys, and treats for environmental enrichment. Animals were anesthetized intramuscularly prior to any procedure and all efforts were made to minimize suffering. All animals were observed twice per day as described below in the “Post-exposure Monitoring” section of these methods. Early endpoint criteria, as also described in the “Post-exposure Monitoring” section below, were used to determine when animals should be humanely euthanized.

### 3.3. Virus

SUDV isolate Boniface was obtained from the USAMRIID repository. This stock was derived from a fatal human case from the 1976 outbreak in Sudan [24]. After isolation from the human case, the virus was passaged once in guinea pigs and four times in Vero cells. The stock was tested and verified negative for endotoxin, mycoplasma, adventitious agents and contamination with other filoviruses. For aerosolization, virus was diluted to an appropriate concentration with Eagle’s Minimum Essential medium with non-essential amino acids (EMEM/NEAA) containing 10% fetal bovine serum (FBS) and 0.1% gentamicin.

### 3.4. Aerosol Exposure

The method used for aerosol exposures was previously described [25]. In brief, the NHPs were anesthetized by i.m. injection with tiletamine/zolazepam (6 mg/kg) or ketamine-acepromazine (9 mg/kg of ketamine and 0.1 mg/kg of acepromazine). Whole body plethysomography was performed under the plane of anesthesia (Buxco Research Systems, Wilmington, NC) for calculation of the respiratory minute volume. Animals were then exposed to SUDV in a head-only aerosol chamber within a class III biological safety cabinet for a time-calculated aerosol exposure. Aerosols were generated by a three-jet Collison nebulizer (BGI, Inc, Waltham, MA) controlled by an automated exposure control system [26]. The average mass median aerodynamic diameters (MMAD) of the generated aerosol particles was 1.4 µm with a geometric standard deviation (GSD) of 2.1, as measured by a Model 3321 Aerodynamic Particle Sizer (TSI, St. Paul, Minnesota). The temperature of the chamber during the spray was 22–28 °C. The steady-state humidity in the aerosol chamber ranged from 35–65%. The generated aerosol was sampled with an all-glass impinger (AGI) containing 10 mL of collection medium (EMEM/NEAA, 10% FBS, 0.1% gentamicin, and 0.001% antifoam A). Immediately after the exposure, AGI samples were analyzed by performing a plaque assay on Vero 76 cells. The inhaled dose was calculated for each NHP by the following formula: Dose V_m_ x t x C_e_, where V_m_=respiratory minute volume, t=duration of the exposure and C_e_=aerosol concentration [25]. The exposure time was calculated based on the minute volume of each NHP so that the same inhaled dose could be delivered to each animal with the dosage group. 1000 pfu is a standard filovirus challenge dose in the field and the original target doses for this study were 1000 and 100 pfu. However, in the first tier of this experiment the dose was 2-fold lower than the intended 1000 pfu target dose. To maintain consistency for the remaining 5 experimental tiersthe target doses were adjusted to 500 and 50 pfu.

### 3.5. Plaque Assay

SUDV samples were titrated in complete EMEM/NEAA supplemented with 10% FBS and 0.1% gentamicin. Dilutions were plated in 6-well dishes containing approximately 100% confluent Vero 76 cells. After a 1 h attachment incubation at 37°C, wells were overlaid with 1% agarose in Eagle’s Basal medium (EBME) (with 10% FBS and 0.1% gentamicin) and returned to the incubator for 7 days. On day 7, a 1% agarose secondary overlay containing 5% neutral red was added and after 2 more days at 37 °C, plaques were counted.

### 3.6. Post-Exposure Monitoring

NHPs were examined and evaluated by study personnel for clinical signs of disease at least two times per day after exposure. Animals were observed for responsiveness and appearance and, for the morning observations, anesthetized with tiletamine/zolazepam (3 mg/kg; i.m) for the remainder of the assessment. The following assessments were made and scores recorded: telemetry device temperature (0 = no change from baseline; 1=↑≥1.5 °C; 2=≥↑2 °C; 20=↓4 °C), responsiveness/appearance (0 = normal; 2 = depression, mild unresponsiveness, becomes active when approached; 5 = head down, tucked abdomen, hunched, flushed face, grimace; 10= moderate unresponsiveness, weakness, persistent prostration but rises when approached; 20=severe unresponsiveness & prostrate), petechial rash (0 = none; 1 = barely visible; 2 = moderate; 3 = widespread), weight loss (0 = no change from baseline; 2 = ↓10%; 3 = ↓15%; 20 = ↓20%), bleeding (0 = none; 2 = blood withdraw site; 3 = rectal), urine (0 = normal; 3 = none), gastrointestinal signs (0 = normal; 1 = little stool; 2 = diarrhea or no stool; 3 = vomiting), appetite (0 = eating; 1 = no food 1 day; 2 = no food ≥ 2 days), breathing (0 = normal; 2 = tachypnea/dyspnea; 3 = labored; 20 = agonal), and dehydration (0 = not present; 2 = moderate; 3 = severe). Animals that met the clinical criteria (as defined in the approved research protocol) were humanely euthanized. The early endpoint criterion for humane euthanasia was a cumulative total score of ≥15. A necropsy was performed on each animal and tissue samples were collected for histological and virological analyses, the results of which will be a topic of a future publication.

### 3.7. Telemetry Data Collection and Analysis

PhysioTel D-70-PCT (TL11M2-D70-PCT) radiotelemetry devices (Data Sciences International (DSI), St. Paul, MN, USA) collected temperature, arterial pressure, lead II electrocardiogram (ECG), heart rate and activity readings for a 4 minute period every hour beginning at least 3 days prior to challenge and continuing up to 14 days post-exposure. Data was recorded by the DataQuest A.R.T 4.1 system (DSI). Pre-exposure temperature data was used to develop a baseline period to fit an autoregressive integrated moving average (ARIMA) model. Forecasted values for the post-exposure period were based on the baseline extrapolated forward in time using SAS ETS (vers. 9.2). Residual changes were determined by subtracting the predicted value from the actual value recorded for each time point. For temperature, residual changes greater than 1.5 degrees were used to compute fever duration (number of hours of significant temperature elevation), fever hours (sum of the significant temperature elevations), and average fever elevation (fever hours divided by fever duration in hours). The diurnal changes in heart rate were assayed by calculating the standard deviation of the heart rate (SD-HR) for each 24 h. 

### 3.8. Clinical Laboratory Analysis

While the NHPs were anesthetized, blood samples were collected from a femoral vein of each animal. Approximately 1.5 mL, less than 1% of the total blood volume, was collected each time in accordance with USAMRIID animal welfare policies. On the day of aerosol challenge (day 0), the samples were collected before exposure to virus; these samples served as a baseline. Each surviving animal had samples collected daily, days 1-10 post-exposure. One rhesus that survived the viral challenge in the low viral dose group also had samples collected on days 12 and 14. Complete blood counts (CBCs) and blood chemistries were analyzed with Beckman Coulter ACT-Diff hematology (Brea, California) and Abaxis Piccolo chemistry (Union City, California) analyzers. Analysis of serum chemistry included the following concentration measurements: glucose (GLU), blood urea nitrogen (BUN), creatinine, uric acid (UA), total calcium (CA), albumin (ALB), total protein (TP), alanine transaminase (ALT), aspartate aminotransferase (AST), alkaline phosphatase (ALP), total bilirubin (TBIL), gamma-glutamyltransferase (GGT), and amylase (AMY).

### 3.9. Real-Time Reverse Transcription Polymerase Chain Reaction (RT-PCR)

RNA was isolated from blood samples using the Ambion MagMAX Viral RNA Isolation Kit following the protocol that is included with the kit (Austin, Texas). SUDV specific primers (F1051 and R1130) (Invitrogen) and p1079S-MGb probe (MGB), as published in Trombley *et al.*, were used to amplify the target (nucleoprotein gene) [23]. The reactions were assembled using the Invitrogen SuperScript One-Step RT-PCR Kit plus bovine serum albumin (Ambion) and run on a Roche Light Cycler (Roche Applied Science, Indianapolis, ID) as described previously [23]. A standard curve was made to correlate the RT-PCR results to standards with known plaque-forming unit (pfu)/mL and the results are reported as pfu/mL.

### 3.10. Statistical Tests

For comparison of mean time-to-moribund condition and temperature variables between groups, t-tests with stepdown bootstrap adjustment were used. Kaplan Meier survival analysis and log-rank tests with step-down Sidak adjustment were used to compare survival curves among groups. Comparison of survival rates among groups was performed with Fisher’s exact tests with step-down bootstrap adjustment. T-tests were performed to see if significant increases or decreases had occurred throughout the disease course. These were 2-tailed paired t-tests. All tests were done for each group, comparing the last day at which all animals in a group were still alive with the baseline taken on day 0 before exposure. In several cases, this value was not significant because animals within the same group progressed at different times, with some animals from a group reaching the criteria for euthanasia days before others. Therefore, t-tests were also performed between the baseline data and the days at which each animal from a group was euthanized. If either test showed significance, this value was reported. Baseline and minimum values for SD-HR were compared for each species using paired t-tests. The days to the minimum SD-HR and the days to moribund condition were also compared for each species using paired t-tests. An alpha value of 0.05 was used as the criterion for statistical significance. 

## 4. Conclusions

Very limited data exists on SUDV infection of NHPs, with several publications describing the infection of one to three cynos as controls for vaccine studies, utilizing i.m. or aerosol routes. Cynos succumbed on days 6-8 by i.m. inoculation and days 7-9 by aerosol inoculation [11,27,28]. A single rhesus control inoculated with SUDV by the i.m. route succumbed on day 17 post-exposure [29]. The macaques in these studies had the presence of a rash, lymphopenia, thrombocytopenia, and increases in ALT, AST and BUN but few other clinical findings were described. Thus, the study reported here, utilizing the aerosol exposure route, represents the first detailed characterization of the disease caused by SUDV in AGMs, cynos and rhesus.

To summarize, exposure to aerosolized SUDV results in a disease course with numerous similarities among AGMs, cynos, and rhesus: with decreased platelets, kidney and liver injury, likely leading to reduced blood volume, followed by altered blood pressure and heart rate in the final stage. Dyspnea and fever develop in all three species. In addition, all three species have increases in WBC and BUN and decreases in lymphocytes, albumin and calcium throughout the disease course. Some important differences include more prominent increases in ALT, AST and ALKP in AGMs. AGMs also had the earliest fever onset, fewest occurrences of petechia and less alteration in heart rate and blood pressure. Cynos, which are typically used in vaccine studies, had shorter survival time on average, the highest frequency of petechia and the greatest alteration of heart rate. In contrast, rhesus, a species often used to test filovirus therapeutics, had the longest average survival and less severe clinical disease. Accordingly, the smallest increases in ALT, AST and ALKP and lowest viral load in the blood occurred in rhesus. The reason for the difference in peak viral load could be worthy of exploration, particularly whether it is related to the longer survival time or the recovery of one rhesus. 

Disturbances in the heart rate and blood pressure coincided with the onset of fever and these changes were more pronounced in rhesus and cynos than AGMs. Increased heart rate and decreased blood pressure could be caused by a variety of factors; in addition to being a generalized response to infection and fever, any vascular leakage or coagulopathy could also be contributory. Perturbations in heart rate coinciding with fever in cynos infected with aerosolized Marburg virus*,* has also been reported [22]. Additionally, changes in blood pressure and increased pulse and respiratory rate are more pronounced in EBOV infected rhesus succumbing to infection than survivors [30].

In all three species of NHP studied, a loss of the diurnal fluctuation in heart rate and reduced heart rate variability, an indicator of poor prognosis in cardiovascular diseases, was observed after viral challenge with SUDV [31]. To the authors’ knowledge, this is the first report of such a change in in ebolavirus infection. Cardiac abnormalities, such as diffuse myocarditis and tachycardia, have been observed in humans infected with MARV and EBOV [10]. However, similar losses of diurnal heart rate fluctuation have been reported previously in both cynos and AGMs after infection with pneumonic plague, suggesting that such a change in heart rate as observed in the present study is not unique to ebolavirus infections [32,33]. 

It is possible that the WBC counts initially increased as a normal host response to infection and that the subsequent decrease was caused by destruction of the WBCs due to direct viral infection of the cells and/or by “bystander apoptosis” of lymphocytes [34]. In another study, rhesus infected with EBOV by the aerosol route developed leukocytosis on day 4 post-exposure with peak counts between ~10,000 and 15,000 cells/µl followed by leucopenia later in infection [13]. Likewise, consistent with the results of this study, lymphocytopenia is typically observed later in the filoviral disease course, including a study of rhesus infected with SUDV by the i.m. route [29]. Another hallmark of filovirus infections is marked coagulopathy [16]. Because of limitations of the blood volume collected, coagulation was not directly measured in this study. However, the significant decreases in platelet numbers that were observed in all three NHP species are consistent with what typically occurs in disseminated intravascular coagulation (DIC). The presence of a petechial rash in many of the animals and hemorrhaging from body orifices and/or venipunture sites in some monkeys were also suggestive of coagulopathy. 

Increases in BUN can be caused by increased production of urea due to protein catabolism associated with fever, necrosis, and/or hemorrhage into the intestinal tract. Both BUN and creatinine are removed from blood by the kidneys and excreted in the urine; this is dependent on adequate blood flow to the kidneys and proper renal function. Although the fevers present in the monkeys in this study may have accounted for some of the increases seen in the BUN, another explanation for the increased concentrations of BUN and creatinine observed in these animals is decreased renal excretion, caused by reduced blood volume due to dehydration and/or virus-induced vascular leakage. Decreased renal excretion is supported by cage-side observations of decreased urine output in this study. 

All of the NHPs in this study had significant hypoalbuminemia that was possibly caused by a combination of decreased hepatic production of albumin and albumin loss through hemorrhage and/or vascular leakage. For both groups of rhesus, the decreases seen in total protein correlated with the decreases in albumin and calcium. However the total protein concentrations in the cynos and AGMs were not as clearly linked to the degree of hypoalbuminemia and it is possible that increased production of acute phase reactant proteins and immunoglobulins by these animals partially offset the decreased albumin and calcium concentrations.

Aerosolized SUDV, reported here, and previously published characterization of aerosolized EBOV and MARV in NHPs, are summarized and compared in Table 3 [7,22]. Interestingly, cynos succumb earliest to either aerosolized SUDV or EBOV and have the greatest decreases in lymphocytes. Increases in WBCs and decreases in platelets occur in aerosolized SUDV, EBOV and MARV infection of each NHP species tested. Liver and kidney damage occur in both SUDV and MARV aerosol infected NHPs, as do blood pressure and heart rate perturbations, although these parameters were not measured in the EBOV study. Overall, the disease course among the three NHP species infected with aerosolized SUDV was similar, albeit with some nuanced differences that were previously highlighted. This, the first characterization of the clinical disease course of aerosolized SUDV in NHPs, can serve as a foundation for testing vaccines and therapeutics. 

**Table 3:** Comparison of aerosol exposure of NHPs to filoviruses.

Virus	SUDV			EBOV [7]			MARV [22]
Species	AGM	Cyno	Rhesus	AGM	Cyno	Rhesus	Cyno
**Survival (d.p.e.)**	8.7	7.7	9.3	8.3	6.7	7.3	8.3
**Fever Onset (d.p.e.)**	3.7	4.5	3.9	5.2	3.3	5.8	5.0
**Rash**	50% (barely visible)	100% (widespread)	50% (moderate)	None	100% (prominent)	100% (prominent)	100%(Mild to moderate)
**Viremia** ** (peak)**	3.5 x 10^7^	1 x 10^8^	5.5 x 10^5^	~8 x 10^7^	~7 x 10^6^	~7 x 10^7^	6 x 10^7^
**Renal **	BUN ↑85%	BUN ↑133%	BUN ↑49%	N.D.	N.D.	N.D.	Fibrin thrombi and acute degeneration
**Liver (↓protein, ↓albumin)**	Yes	Yes	Yes	N.D.	N.D.	N.D.	N.D.
**Liver **	1.3:1 (AST:ALT)	3.1:1 (AST:ALT)	3.8:1 (AST:ALT)	N.D.	N.D.	N.D.	Mild lesions
**WBCs**	↑27%	↑36%	↑57%	↑~150%	↑~25%	↑~200%	↑~92%
**Lymphocytes**	↓62%	↓73%	↓39%	↓46%	↓76%	↓39%	Reported # not %
**Platelets**	↓42%	↓44%	↓44	↓56%	↓19%	↓50%	↓~28%
**Heart Rate (Tachycardia)**	Mild	Severe	Moderate	N.D.	N.D.	N.D.	Present
**Blood Pressure (Perturbation)**	Yes	Yes	Yes	N.D.	N.D.	N.D.	N.D.
